# A glance at black cumin (*Nigella sativa*) and its active constituent, thymoquinone, in ischemia: a review

**DOI:** 10.22038/ijbms.2018.31703.7630

**Published:** 2018-12

**Authors:** Zahra Oskouei, Maryam Akaberi, Hossein Hosseinzadeh

**Affiliations:** 1 Department of Pharmacodynamics and Toxicology, School of Pharmacy, Mashhad University of Medical Sciences, Mashhad, Iran; 2 Department of Phamacogenosy, School of Pharmacy, Mashhad University of Medical Sciences, Mashhad, Iran; 3 Pharmaceutical Research Center, Pharmaceutical Technology Institute, Mashhad University of Medical Sciences, Mashhad, Iran

**Keywords:** Black cumin, Brain, Cardiovascular, Ischemia, Nigella sativa

## Abstract

**Objective(s):**

Black cumin (*Nigella sativa*) belonging to Ranunculaceae family has a long history of medicinal use in various folk and traditional systems of medicine, including Iranian traditional medicine (ITM). These valuable medicinal seeds have been used traditionally against a variety of diseases such as dyspepsia, diabetes, headache, influenza and asthma. In addition, several scientific investigations have reported the therapeutic properties of *N. sativa *and thymoquinone (TQ), one of the most important constituent of black cumin, for treatment of a large number of diseases, including ischemia. As there is no comprehensive review study about the anti-ischemic activity of black cumin and its mechanism of action, in the current study, we aimed to review the anti-ischemic activities of *N. sativa* and TQ in different organ-related disorders.

**Materials and Methods:**

We searched the words *N. sativa* or black cumin and ischemia in the combination of related organs through available databases including Scopus, Web of Science, and Google Scholar.

**Results:**

Several studies were found reporting the anti-ischemic activity of black cumin and its active constituent on different organs including brain, kidneys, heart, and liver. Black cumin exert its beneficial effects as an antioxidant, anti-inflammatory, anti-apoptosis, and anti-necrosis agent through inhibition of growth factors, biochemical and oxidative stress markers and regulating gene expression.

**Conclusion:**

Thus, *N. sativa *could be a potential candidate for treatment of ischemia related disorders in key organs such as brain, liver, digestive system, kidney, and heart. To figure out the exact mechanism of action, further investigations are proposed in this regard.

## Introduction

**Figure 1:**
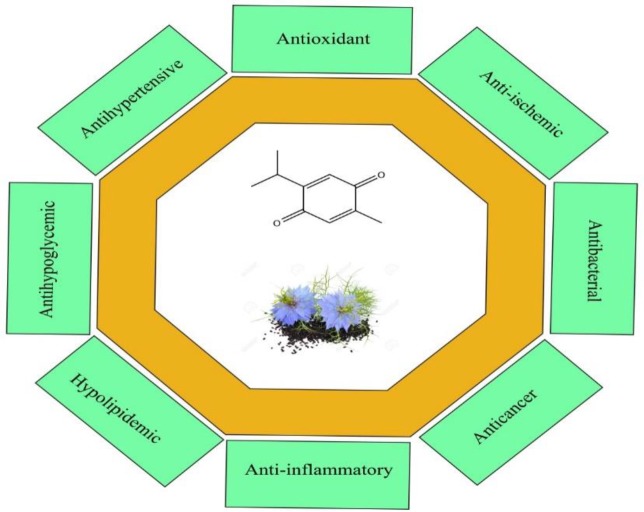
The chemical structure of thymoquinone (TQ) and a schematic diagram of the pharmacological activities of *Nigella sativa *and TQ

The date of using plants as medicines by humans is not certain but the historical findings show that at least it dates back nearly 4000 years ago. Interest in medicinal plants is due to newly observed valuable activities of natural drugs which lead to the growing interest in the field of natural product research. In addition, this interest has been increased because of significant adverse effects of synthetic drugs leading to substitution of natural products for the treatment of various diseases in the past decade (1, 2). *Nigella sativa *belonging to Ranunculaceae family is a dicotyledonous plant and is mostly distributed in Southern Europe, North Africa, and Asia Minor. This amazing plant has a rich religious and historical background (3). It is a bushy plant with white or pale to dark blue flowers. The seeds are the parts of the plant which is used in herbal medicines; when the fruit capsules open, the black seeds are dispersed in the air (4). *N. sativa *has been used for treatment of various diseases from ancient times. In the Holy Bible, it was identified as the curative black cumin and Hippocrates described it as the Melanthion and Dioscorides as the Gith of Pliny (5). This plant is also known as the black seed and black cumin. Historically, this plant has been used for the treatment of toothache, flatulence, as a choleretic, anti-spasmodic and uricosuric (6), nasal congestion, hypertension, obesity, back pain, amenorrhea (7), infections, and intestinal worms (3, 4, 8, 9). Studies have revealed that black cumin and its bioactive constituent, thymoquinone (TQ) have hypoglycemic (10, 11), hypolipidemic (12), antioxidant (13, 14), anti-inflammatory (15), anti-tumor, anti-convulsant, and anti-tussive activities (16-19) (Figure 1). In addition, it is a protective agent against natural or chemical toxins (20), an anti-aging agent (21) and a protective agent against ethanol induced toxicity (22). 

**Figure 2:**
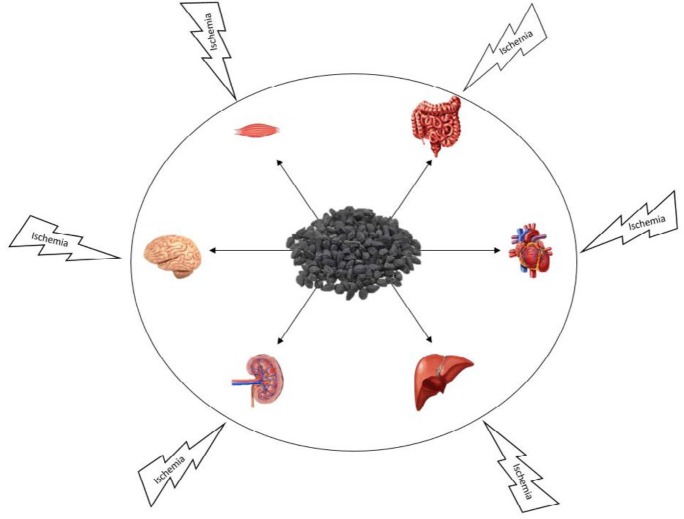
Protective effects of *Nigella sativa* against ischemia in different organs

Ischemia-reperfusion injury (IRI) is a complex of intracellular chemical operation and inflammatory response. Reduction in cellular energy (ATP) content, as one of the mechanisms for IRI, leads to destroy cellular ion homeostasis with activation of hydrolases and damage of elective permanence of cell membranes and cause of decrease in organ function after transplantation and limit in survival of transplanted organs (23) (Figure 2). IRI damages O_2_-dependent cells of tissues and organs such as brain, liver, kidney, intestine and heart; mitochondrial oxidative phosphorylation can provide energy need of these cells which cannot be supplied by anaerobic glycolysis alone (24, 25). A reduction in mitochondrial energy leads to decrease in pH of the cell, increase in the anaerobic glycolytic level, and release of H^+^ from the lysosomal; Finally, the increasing flow rate of cytosolic Na^+^ and Ca^2+^ destroys cellular ion homeostasis (23).

In this study, the anti-ischemic activity of *N. sativa* and TQ on different organs including brain, liver, kidney, etc. is reviewed.

## Methods

The data were collected by searching through scientific databases PubMed, Scopus, Google Scholar, and Web of Science. *N. sativa*, black cumin, and ischemia were the main keywords used as search terms. All kinds of relevant articles, abstracts, or books were included. Furthermore, the reference lists of key papers for further leads were searched. Both *in vivo* and *in vitro* studies were included to this investigation. No time limitation was considered in this review. 

***Phytochemicals***

The phytochemicals found in black cumin can be divided in two categories; volatile compounds and non-volatile metabolites.

*Volatile compounds*

The biological activity of *N. sativa* is mostly attributed to its volatile oil compounds especially TQ (26). Black cumin contains 0.40–0.45 %w/w of a volatile oil including 18.4–24% TQ, and 46% other monoterpenes such as ρ-cymene, thymol, carvacrol, ρ-cymene and α- and β-pinene (27).

The essential oil of black cumin is usually obtained by hydrodistillation, however other methods such as microwave-assisted extraction and super critical fluid extraction is also used (28, 29). Botnick *et al*. has proposed a method for the isolation of black cumin essential oil. For this purpose, the seeds were ground with a mortar and pestle after they were frozen in liquid N_2_. The extraction of the volatile fraction was made by adding a 3 to 1 ratio (v/w) of tert-butyl methyl ether (MTBE). After a short vortex, the ground seeds were shaken for 2 h at room temperature (25-30 ^o^C) for extracting volatile fraction (30). 

*Non-volatile compounds*

The other category of secondary metabolites found in *N. sativa* is non-volatile compounds. Alkaloids are one of the most important bioactive constituents of *N. sativa*; the seeds have two different forms of alkaloids: isoquinoline alkaloids such as nigellicimine and pyrazol alkaloids including nigellidine and nigellicine. Moreover, saponins, fatty acids, carbohydrates, fixed oils, proteins, and phenolic compounds such as flavonoids, have been reported from this plant (31, 32). The plant is also a source of calcium, iron, and potassium (33).

***Pharmacological activities***

A large number of activities have been reported for *N. sativa *and its bioactive constituents including anti-convulsant (34, 35), hypotensive, anti-nociceptive, uricosuric, choleretic, anti-fertility, anti-diabetic, anti-microbial and antibacterial (31, 36). *N. sativa* have also shown analgesic, anti-inflammatory (37), anti-cancer (38), anti-histaminic (39), and neuroprotective (40, 41) properties (Figure 1). Moreover, *N. sativa* and TQ had a potential role in the management of metabolic syndrome and cardiovascular diseases risk factors including high blood pressure, obesity, dyslipidemia and high blood glucose (42). Among these various activities, black cumin has strong effects against ischemia reperfusion (IR) injury on various organs. In the following paragraphs, the anti-ischemic properties of *N. sativa* and its bioactive compound namely TQ on various organs will be discussed.

***Anti-ischemic activities***

*Brain*

Ischemic brain damage as one of the most important causes of adult disability (43), stops the blood flow completely and produces inadequate delivery of oxygen to the brain tissue that lead to a decrease in glucose and adenosine triphosphate (ATP) levels through anaerobic glycolysis. Lactic acidosis produced by ATP discharge can reduce energy and operating cofactor redistribution of fast ions in neuronal and glial cell and depolarization occurs on cell membrane (44). Events that generally occur during ischemia including brain ischemia are: 1) The loss of membrane lipid by lipolysis and by radical-mediated peroxidation of poly-unsaturated fatty acids during reperfusion; 2) Protein synthesis in the brain is inhibited at the translation initiation and prolonged arrest of protein synthesis in elective vulnerable neurons during post-IR; 3) Apoptotic mechanisms, activation of proteolysis, and activation of endonucleases lead to injury of critical molecules and destroy cellular repair processes during reperfusion. 

Antioxidants have essential roles in recovery of damaged brain cells (45). They regulate initiation of translation by various mechanisms including activation of initiation factors, inhibition of apoptosis, and improve the restoration of injured organelles at a fundamental level by signal transduction mechanisms involving growth factors (45). Studies show that different extracts of *N. sativa* and TQ have antioxidant and neuroprotective activities in cerebral ischemia (46, 47). *In vitro* and *in vivo* investigations have reported an increase in reduced glutathione (GSH), superoxide dismutase (SOD), and catalase levels which are indicative of cerebral ischemia. In addition, after administration of aqueous and hydroalcoholic extracts of *N. sativa*, a decrease in malondialdehyde (MDA) level and an inhibition in lipid peroxidation have also been reported in several studies (48). For example, Al-Majed *et al.* have reported that oral administration of TQ could reduce MDA level and elevate GSH and SOD levels in transient forebrain ischemia in the rat hippocampus (49). Hosseinzadeh *et al*. confirming the results of the previous study, reported the protective effects of *N. sativa* seeds oil and TQ on cerebral ischemia in a rat model. The results exhibited the beneficial effects of *N. sativa* on lipid peroxidation during global cerebral IRI (50). In some studies, the protective activity of TQ against hippocampal neurodegeneration has been investigated (51, 52). Remarkable changes were observed in biochemical factors after the treatment with TQ (5 mg/kg, PO); a significant decrease in MDA content, lipid peroxidation, and an increase in GSH level, SOD and catalase activities (52). Edema is one of the major consequences of both peripheral and intracellular global IRI which happens in pyramidal and interstitial cornu ammonis cells as well as supportive neuronal tissue such as cytoplasmic glial cells (53). In a rat model, different doses of *N. sativa *extracts (1, 10, and 50 mg/kg) were injected to the animals. The results showed that the extract has a significant ability to protect neuronal tissue of hippocampus and prevents edema in a dose-dependent manner (52). Soleimannejad *et al*. showed that hydroalcoholic extract of *N. sativa* (20 mg/kg) could increase the markers of cerebral angiogenesis after global ischemia of brain in rats (54). Accordingly, a significant decrease in brain edema and infarct volume, an increase in gene expression of vascular endothelial growth factor (VEGF) and hypoxia-inducible factor (HIF), and a decrease in the expression level of matrix metallopeptidase-9 were observed. The inhibitory effect of black cumin against cerebral IRI is also confirmed by *in vitro* and *in vivo* studies (52, 55). Effects of various extracts of *N. sativa *such as aqueous, hydro-alcoholic, chloroform and petroleum ether refers to decline lipid peroxidation and increase glutathione and antioxidant enzymes, (superoxide dismutase and catalase) in middle cerebral artery occluded rats (56, 57). Black cumin extract also could reduce hippocampal neurons swelling and astrocytes loss after global cerebral IRI in rats. *N. sativa *extract (1 and 10 mg/kg) also could prevent the edema of pyramidal neurons (52). Moreover, pre- and co-administration of TQ (20 mg/kg) has also been reported to decrease brain ischemia induced by neurotoxic effects of lead in rats through the same mechanism of action (58). 

*Kidneys*

Renal ischemia injury is one of the ordinary difficulty that occurs during transplantation, retail nephrectomy, cardiopulmonary bypass, or hydronephrosis that may lead to kidney dysfunction (59). This problem may also happen in the context of cardiac arrest during recovery vascular operation which is an important cause of renal cell death, renal failure, and delayed graft function (60, 61). There are substantial number of contributor to renal ischemia including anoxia, free radicals and inflammation responses (62, 63). ROS and stress oxidative lead to structural and functional damages (59) including detrimental effects in endothelial, glomerular, mesangial, and tubular epithelial cells (particularly S3 segment of proximal tubule) (64, 65), a reduction in the impact of ion pump activity and DNA damages such as DNA inhibiting transcription and repair (66, 67) causing acute kidney injury, acute rejection, acute tubular necrosis, chronic allograft failure, and acute renal failure (63, 68, 69). *N. sativa* has remarkable beneficial effects on renal ischemia mostly because of its immunomodulatory and antioxidant properties (70). Aqueous and ethanol extract of *N. sativa* (1.6 g/kg) as well as TQ are reported to have protective effects on renal ischemia by decreasing MDA content of renal tissue and increasing the renal thiol content. They can also diminish free radical-mediated lipid peroxidation, DNA damage and reduce thiobarbituric acid reactive substances (TBARS) levels (71-75). It is also reported that pre- and post-treatment of *N. sativa* seed oil can diminish kidney oxidative stress signs and tubular necrosis score after IRI (76). Several other studies confirm the protective effects of *N. sativa* on renal ischemia via decreasing oxidative stress markers. In 2008, Omer Bayraket *et al*. investigated the protective effect of *N. sativa *against IRI in rat kidneys. They demonstrated that pre- and post-treatment with *N. sativa *oil has protective effects on IRI through improving renal failure including a decrease in MDA levels, nitric oxide (NO) concentration and protein carbonyl content (PCC) in serum and tissue, as compared to the control group (77). 

Pretreatment with *N. sativa* significantly can decrease the levels of creatinine, blood urea nitrogen, and uric acid as well as the water intake and urinary excretion. In addition, the ratio of renal to body weight (the renal-body weight degree) is diminished and the index of histopathological damages such as cellular vacuolation, interstitial edema, hyperemia, hemorrhage, tubular necrosis, and glomerular changes are improved (78). Studies show that *N. sativa* not only affect biochemical parameters, but also it has influences on gene and protein expression. TQ decreased cytochrome P450 3A1 (CYP3A1) and spermidine/spermine N-1-acetyl-transferase (SSAT) gene expression that were up-regulated in ischemia in the kidneys (79). In another study, confirming the protective effects of TQ, pre- and post- administration of TQ could result in a significant improvement in the tubular renal cells and hemodynamic functional parameters and in debilitation of the gene expression of some of the pro-fibrotic cytokines and pro-inflammatory, namely tumor transforming growth factor beta 1 (TGF-β1), the type-1 inhibitor of plasminogen activator (PAI) and necrosis factor alpha (TNF-α). In addition, TQ reduced the expression of some of the markers of acute renal injury such as neutrophil gelatinase-associated lipocalin (NGAL), kidney injury molecular 1 (KIM-1) and neutrophil gelatinase-associated lipocalin (NGAL) (80). Moreover, *N. sativa* could decrease the degenerative changes in the glomerulus and tubules of kidney cortex and the positivity of NF-kappa-B p65 subunit (NFkB p65) in the renal cortical tissues (81).

**Table 1:** Anti-ischemic activity of *Nigella sativa* and its active constitute TQ on different organs

**Ischemic condition**	**Study design**		**Result**	**Ref.**
**Transient global cerebral ischemia**	Five N.G Wister albino rats i.p administration		Reduction in MDA and elevation in thiol (SH)	(137)
**Focal hippocampus ischemia**	Wister rats (N=30), (1, 10, and 50 mg/kg, i.p)		Prevent intracellular edema of interneurons in 50 mg/kg group significantly compared to the sham group	(49)
**Transient global cerebral ischemia**	TQ (2.5, 5 and 10 mg/kg) and *N. sativa* oil (0.048, 0.192 and 0.384 mg/kg, i.p), Rats		Dose-dependent reduction in the free radical-mediated lipid peroxidation as indicated by a decrease in the MDA levels	(138)
**Renal ischemia**	Wistar albino rats, 0.3 ml gastric tube		Significant reduction in serum and tissue MDA, NO and PCC and subsequent elevation in anti-oxidant power	(69)
	Rats, 150-300 mg/kg, i.p		Dose-dependent reduction in MDA level and an elevation in total Thiol content and glutathione peroxidase Decreases in oxidative DNA damage	(70)
**Hepatic ischemia**	Wister rats, Aqueous extract (0.7, 1 and 1.6 g/kg) ethanol extract (0.7, 1 and 1.6 g/kg) and TQ (2.5, 5 and 10 mg/kg, i.p.)		No significant effect on the radical-mediated lipid peroxidation (aqueous extract) A significant reduction in the MDA levels (ethanol extract, 1.6 g/kg) TQ in doses 5 and 10 mg/kg reduced free radical-mediated lipid peroxidation significantly, by a decrease in the MDA levels TQ pretreated groups, a reduction in TBARS levels, ethanolic extract only in upper dose increased anti-oxidant power	(68)
	Wister-albino rats, TQ (5, 20, and 50 mg/kg)		A decrease in plasma ALT, AST, and LDH levels A significant decrease in pathological changes and histological tissue damages	(89)
**Heart ischemia**	Wistar albino rats (n=44), TQ (10 mg/kg, i.p)		A significant decrease in infarct size Protective effect against reperfusion-induced arrhythmias	(133)
	Wistar rats (20 mg/kg orally)		No significant change in biochemical and histological parameters A significant decrease in the heart weight/body weight ratio Improvement in myocardial SOD, GSH activity, A decrease in the level of MDA, AST, ALT, LDH, and CK (the diagnostic markers of myocardial) A significant decline in the myocardial levels of IL-1, IL-6, and TNF-α	(139)
**Intestinal ischemia**		Rats, TQ (50 mg/kg i.p.)	Dose-dependent reduction in MDA An elevation in SOD activity, GSH-Px activity Reduction in the number of apoptotic cells	(112)
**Skeletal ischemia**		Rats, aqueous extract (1, 1.5 and 2 mg/kg), ethanol extracts (1.6, 2.4 and 3.2 g/kg, i.p.)	Decrease in MDA level, Increase SH level Increased anti-oxidant power (FRAP value)	(45)

*Liver*

Another organ that may be influenced by ischemia is liver. Hepatic IRI is an important factor associated with a high morbidity and mortality that lead to destructive effect on metabolic and structural functions (82). Generally, it happens in conditions with low blood flow to the liver resulting in insufficient perfusion such as liver resections and transplantation (83), cardiogenic and hemorrhagic shock, surgical cutting, cardiovascular or laparoscopic surgery and abdominal compartment syndromes (84, 85). IRI of the liver is complex and multifactorial pathophysiological process involving the generation of numerous ROS and reactive nitrogen species (RNS) (86-88). In the early stages of reperfusion in the liver, cellular edema, vacuolization, endothelial cell disruption, neutrophil penetration, and hepatocellular necrosis may occur (89). To decrease these mechanisms and phenomena in hepatic IRI, several investigations have been established. In this regard, natural products play important roles. The activity of *N. sativa* as a potential medicinal plant against liver ischemia has been studied by different research groups (91, 92). For instance, it is reported that TQ has a strong positive effect against liver IRI, and can down-regulate the expression of CYP3A1 and SSAT gene in the ischemic liver (79). The efficacy of *N. sativa* to postpone progression in chronic liver diseases should be considered as preventive medicine in patients with hepatic disorders (91). 

TQ could also decrease the adverse effects of ROS in IRI condition by increasing catalase activity which shows a potent protective effect on liver tissue. In the liver ischemia, apoptotic cell death is activated and as a consequence, the levels of caspases 8, 9, and 3 are increased. This study has shown that TQ (50 mg/kg) with anti-apoptotic effects could reduce the activities of caspases 8 and 3 via direct and indirect mechanisms. TQ could deactivate caspase 8 and inhibit cytochrome c release from mitochondria and finally inactivate caspase 3. Decrease in cytosolic cytochrome c rate lead to inhibition of lipid peroxidation measured as MDA liver tissue content. In addition, TQ could decrease pro-apoptotic bax protein expression and increase significantly the anti-apoptotic Bcl-2 protein. NF-κB signaling pathway is one of the multiplex apoptotic pathways activated by TNF-α; one of the anti-ischemic activity for TQ is reduction the expression of TNF-α and NF-κB in hepatic I/R models (92, 93). Moreover, administration of TQ (5, 20, and 50 mg/kg) could decrease both aspartate transaminase (AST) and alanine transaminase (ALT) activities as compared to the untreated group.

*Other organs*

Although most of the studies have focused on the protective activities of *N. sativa* against renal, hepatic, and brain ischemia, there are several reports about the anti-ischemic properties of this plant extract and its major component TQ on other important systems and organs. In the following paragraphs, you can find some investigations on the anti-ischemic activity of black cumin in digestive, and cardiovascular systems, as well as skeletal muscles. 

Digestive system (Intestine)

Several conditions lead to intestinal IRI and numerous damages to the intestine, for instance, shock, incarcerated hernia, cardiopulmonary bypass, midgut volvulus, necrotizing enterocolitis, multiple traumas, and sepsis (94-96). Moreover, mesenteric ischemia caused by different kind of factors such as endogenous vasocontractile substances may lead to develop intestinal IRI (97). Interestingly, mesenteric IR lead to many damages such as hepatic and renal injuries (98). Iglesias *et al.* demonstrated that mesenteric IRI causes acute pulmonary edema and increase the pulmonary microvascular permeability to fluids and proteins (99). Intestinal IR damages lead to detrimental events such as systemic inflammatory response, increasing NO and poly morphonuclear lymphocytes (PMNL), and production of ROS and RNS (100-102). For example, Horton and White reported that production of ROS and lipid peroxidation in cardiac cell membranes play a significant role in cardiac dysfunction after intestinal IRI in rats (103). This multiple organ dysfunction syndrome is related to an interference in energy metabolism, oxygen radicals, overload of intracellular calcium, endothelial cell damage, and leukocyte adhesion (104-106). TQ as a potent antioxidant can suppress intestinal IR damage and decrease oxidative stress. TQ can decrease MDA, SOD, and erythrocyte glutathione peroxidase (GSH-Px)-GSH-Px levels in IR-damaged intestinal tissue (107). El-Abhar *et al*. have shown that black cumin can improve the antioxidant conditions due to an increase in mucin content of the gastric mucosa (90). Black cumin oil has also a protective effect against intestinal ischemia through inhibition the release of leukotrienes and histamine from mast cells (102, 105). Anti-apoptotic effect is reported to be an important protective mechanism of *N*. *sativa* against intestinal ischemia (108-113). Al Mofleh *et al*. showed pathologically that *N. sativa* can prohibit gastric ulcer formation induced by necrotizing factors (114). Treatment with TQ improved pathology and significantly decreased the number of TUNEL-positive cells (115, 116). Studies show that *N. sativa* have protective effect against ischemia-induced gastric mucosal ulcer (107). 

Skeletal muscle

IRI in skeletal muscle, as an important cause of morbidity and mortality in populations, motivate an inflammatory response in the affected muscles (117). Blaisdell *et al.* reported that there are substantial numbers of mortality resulting from several system organ failures in severe cases of limb ischemia (118). It is characterized by a number of detrimental phenomena in tissue, such as cell elevation penetrance in the microcirculation, edema, mitochondrial electrolytic change, increased cytosolic free calcium concentration, decrease of membrane phospholipids in ischemic cells and releasing cytotoxic ROS and simulate an inflammatory response (119, 120).

Studies have revealed that hindlimb ischemia in a Wistar rat model is produced by clamping the common femoral artery and vein mitochondrial electrolytic changes, cell edema, increased permeability in the microcirculation, cytosolic calcium overload, ROS generation, decrease in membrane phospholipids (118, 121). Hosseinzadeh *et al.* have investigated the effects of *N. sativa* and its constituent TQ in decreasing skeletal muscle during ischemia. The results of this study show that after treatment with TQ, MDA content is decreased and GSH level is increased significantly (122). In a rat model, pre-administration of *N. sativa* seed aqueous (1, 1.5 and 2 g/kg) and ethanol (1.6, 2.4 and 3.2 g/kg) extracts intraperitoneally lead to a decrease in MDA levels, an increase in antioxidant capacity (FRAP value) of muscle homogenate samples, and a significant elevation in thiol (SH) concentration, as compared with control-ischemic group (48). It is reported that the administration of TQ plus alpha-tocopherol may strongly protect muscle and nerve tissues against IRI due to their synergistic effects (123). These compounds together could have protective effects on the sciatic nerve and femoral muscle as a result of lower limb IRI. They could significantly decrease the levels of MDA, interleukin-6 (IL-6), and neuronal nitric oxide synthase activity of nerve tissues and increase the level of GSH. By recording intramuscular electro myograph (EMG) signals, it is reported that TQ could increase the muscles activities after IRI in comparison to IR control group (119). 

Heart

Myocardium ischemic can trigger a series of deleterious phenomena such as myocardial damage and life-threatening ventricular arrhythmias. Following the reperfusion of the ischemic myocardium, ROS products lead to decreases in antioxidant activity, generation of lethal ventricular arrhythmias and tissue injury. Studies show that TQ plays an important role on myocardial IRI and reduce the infarct size and suppresses arrhythmia scores, ventricular tachycardia and the incidence of ventricular fibrillation. Intraperitoneal administration of TQ (10 mg/kg) seems to be effective in preserving myocardial IRI induced lethal ventricular arrhythmias in anesthetized rats (124-126). A great number of research projects have shown that antioxidants with suppressive effects could delimit infarction rate and decrease myocardial dysfunction and decelerate development of myocardial infarction (MI) (127, 128). ROS play an important role in the pathophysiology of MI (129, 130). Moens *et al*. in 2005 noted that production of ROS after ischemia and reduction in antioxidant task lead to tissue dysfunction and produce ventricular arrhythmias (131). Various herbal antioxidants have a beneficial effect against myocardial IRI (132-135). TQ could reduce MI by affecting the antioxidant condition and decreasing the ROS levels. For instance, TQ (10 and 20 mg/kg) showed a protective effect in myocardial IR damage in a rat model (136). In another study, it is reported that chronic treatment (3 months) of TQ lead to reduction of oxidative stress in MI and help to maintain the activity of antioxidant enzymes in isoproterenol-induced MI rat model (137). TQ could also reduce heart rate and arterial blood pressure (137). *In vitro* studies have shown that TQ has cardiovascular activity, and regulates arterial force and rate of constriction intercede by blockade of voltage gated Ca^+2^ channels (138). 

Ovary

Atasever *et al.* have studied the *N. sativa* oil on ovarian oxidative damage following IRI, using a rat model (139). They did not observe a significant improvement in *N. sativa* oil-treated group in comparison to control groups. Thus, they proposed further studies to confirm or reject the data.

## Conclusion

All findings discussed above indicate that *N. sativa* and its active constituent TQ have strong effects against IRI on various organs, including brain, liver, digestive system, kidney, skeletal-muscle system, and heart. Black cumin has a long reputation in traditional medicine and in recent years it has been used for treatment of several disorders without any reported side effects. Therefore, this plant can be a valuable agent for ischemia problems. Thus, further complementary studies are proposed in this regard. 

## Conflicts of Interest

The authors have no conflict of interest to declare.
